# Venous Thromboembolism in Hospitalized COVID-19 Patients: Systematic Review

**DOI:** 10.2196/22768

**Published:** 2020-09-01

**Authors:** Kade Birkeland, Raymond Zimmer, Asher Kimchi, Ilan Kedan

**Affiliations:** 1 Cedars-Sinai Medical Center Los Angeles, CA United States; 2 Smidt Cedars-Sinai Heart Institute Beverly Hills, CA United States

**Keywords:** VTE, COVID-19, anticoagulation, SARS-CoV-2, review, heart, morbidity, hospital, incidence, treatment, incidence

## Abstract

**Background:**

Coagulopathy associated with COVID-19 infection and venous thromboembolism (VTE) have emerged as significant contributors to morbidity among patients infected with SARS-CoV-2.

**Objective:**

We performed a systematic review to estimate VTE incidence in hospitalized patients and to analyze characteristic factors in the VTE cohort.

**Methods:**

We searched PubMed and Google Scholar using specified title search terms “SARS-CoV-2” or “COVID-19” and “venous thromboembolism” and “anticoagulation” among others to identify peer-reviewed journal articles published between June 22, 2019, and June 22, 2020. Data were systematically extracted and synthesized using Microsoft Excel for analysis. The main outcome was VTE incidence, and measures included patient characteristics, anticoagulation, and clinical outcomes with assessment for associations.

**Results:**

In total, 14 studies were included comprising 1677 patients. Most patients (n=1306, 82.4%) received anticoagulation (either VTE prophylaxis or treatment). VTE incidence was 26.9% (SE 3.1; 95% CI 20.8-33.1) and was correlated with systematic screening (r^2^=0.34, *P*=.03) and study duration (r^2^=–0.33, *P*=.03). D-dimer was higher for the VTE cohort (5.62 [SD 0.9] vs 1.43 [SD 0.6]; *P*<.001). Odds of VTE were higher at the intensive care unit (odds ratio [OR] 6.38, 95% CI 3.67-11.11; *P*<.001) but lower with anticoagulation (OR 0.58, 95% CI 0.36-0.92; *P*=.02).

**Conclusions:**

Despite the utilization of background anticoagulation, VTE incidence was historically high. Future studies are needed to provide additional data to guide optimal VTE prophylaxis and diagnostic strategies.

## Introduction

Coagulopathy is an impairment in the blood’s ability to form clots. Coagulopathy associated with COVID-19 infection has emerged as a significant contributor to morbidity among those infected with the illness. Although the incidence of coagulopathy is unknown, COVID-19–related coagulopathy has been described as having a unique hemostatic signature [1-3]. Typically reported abnormalities include a mildly prolonged prothrombin time/international normalized ratio (PT/INR) as well as an activated partial thromboplastin time (aPTT), mild thrombocytopenia, and elevated D-dimer and fibrinogen [3,4]. Generally, COVID-19–associated coagulopathy results in a prothrombotic state and is nonconsumptive or progresses to hemorrhage [5]. As such, observational studies and autopsy reports have focused on characterizing thrombotic complications, including venous thromboembolism (VTE).

Potential approaches to treatment are continuing to develop with increasing experience with COVID-19 infection. Institutions have described updated VTE prophylaxis protocols aimed to more aggressively prevent clots [1,2,6]. For example, some describe utilization of standard dose VTE prophylaxis with unfractionated heparin (eg, unfractionated heparin 5000 units by subcutaneous injection twice daily or three times daily) or low molecular weight heparin (eg, enoxaparin 40 mg by subcutaneous injection every 24 hours) for all patients hospitalized with COVID-19 infection rather than based on risk assessment. Others deploy anticoagulation regimens that are higher intensity compared to standard dose VTE prophylaxis. For example, high dose, low molecular weight heparin (eg, enoxaparin 40 mg by subcutaneous injection every 12 hours) or empiric treatment anticoagulation (eg, unfractionated heparin by intravenous infusion; enoxaparin 1 mg/kg by subcutaneous injection every 12 hours) for high-risk patients, which is variably defined (eg, intensive care unit [ICU] level of care, clinical deterioration, rising d-dimer) and based on historical data in other high-risk populations (eg, bariatric surgery, third trimester pregnancy) [1,2,6]. Also, utilization of specific anticoagulation products will vary by country depending on regulatory approval and availability. In fact, early experience indicates that high-risk patients (eg, sepsis-induced coagulopathy score ≥4 or D-dimer >3 times the upper limit of normal [ULN]) are most likely to benefit from VTE prophylaxis [7]. However, evidence-based treatment protocols are needed to further improve in COVID-19 patients with VTE [1,2].

The mechanism of COVID-19–associated coagulopathy is not fully understood. However, the intense and sustained cytokine-mediated inflammatory response to SARS-CoV-2 infection is likely etiologic. As an example, elevated fibrinogen levels have been found to be associated with elevated interleukin-6 (IL-6) levels, while D-dimer also rises in parallel with C-reactive protein (CRP) [8]. Furthermore, inflammatory markers directly activate the clotting system as does tissue hypoxia, both on top of direct endothelial cell injury and subsequent dysfunction by SARS-CoV-2 cellular entry [7,9].

Serologic markers may be associated with severity of infection and may also be predictors of increased morbidity and coagulopathy. For example, D-dimer is a biomarker that increases as a result of thrombosis (eg, microvascular thrombosis, deep vein thrombosis [DVT], or pulmonary embolism [PE]) or systemic activation of hemostasis (eg, disseminated intravascular coagulation) and is associated with severe COVID-19 and mortality [10]. Tang et al [4] demonstrated that D-dimer elevation on admission and rising to at least 3-4 times ULN over the course of the hospital stay was associated with increased mortality. Other hemostatic markers such as PT and aPTT prolongation, elevated fibrinogen degradation product, and low platelets have also been associated with severe COVID-19 and mortality [4,11]. Governing bodies have recommended that D-dimer, PT, platelets, and fibrinogen be measured in patients with COVID-19 infection for risk stratification and prognosis [1,2]. 

We performed a systematic review of VTE in the setting of patients hospitalized with COVID-19 infection and summarized the potential treatment effects in VTE management in these patients. Our aim was to estimate the observed incidence of hospitalized VTE patients and analyze patient characteristics in the VTE cohort. 

## Methods

We performed a systematic literature search in PubMed with the title search terms “COVID-19” or “SARS-CoV-2” or “Novel Coronavirus 2019” and “venous thromboembolism” or “deep vein thrombosis” or “pulmonary embolism” or “thrombosis” or “thromboembolic” or “anticoagulation” or “heparin” or “thromboprophylaxis” to identify primary research studies that report the rate of VTE in patients hospitalized with COVID-19 infection who are treated with standard dose pharmacologic VTE prophylaxis, high dose pharmacologic VTE prophylaxis, treatment dose anticoagulation, no anticoagulation, or no documentation. A supplementary search was performed on Google Scholar using the same search terms and journal article references were reviewed to identify additional studies. Studies of adult populations that were published in a PubMed peer-reviewed journal from June 22, 2019, to June 22, 2020 were included for review. Data were collected for each included study design, population studied, VTE event rate, VTE diagnostic strategy, VTE prophylaxis or treatment strategy, hemostatic lab abnormalities, and clinical outcomes including ICU level of care and survival. 

We excluded studies with arterial thrombosis, myocardial infarction or ischemic stroke, pediatric and fetal populations, and reviews, case reports, letters to the editor, or any study that had not yet undergone peer review. Clinical outcomes data for the included studies were pooled, and we conducted a systematic review and meta-analysis with a random effects model to measure a single group summary for VTE incidence as our primary outcome [12]. Confidence intervals were determined by the adjusted Wald method. Secondary outcomes included a single group summary for mortality with the same methodology as the primary outcome and patient demographics with clinical characteristics using descriptive statistics with weighted mean and weighted standard deviation. Assessment of variables associated with VTE incidence was conducted using univariate linear regression and multivariate linear regression. Assessment of binary variables associated with VTE occurrence was conducted using multiple logistic regression. Estimation of differences in continuous variables between patients with VTE and patients without VTE was conducted using the Z test (two samples for weighted means with weighted variance). Data were compiled using Google Sheets (Google LLC) and Microsoft Excel (Microsoft Corp).

The Cedars-Sinai Hospital Institutional Review Board requirement for approval was waived as this is a systematic literature review.

## Results

**Figure 1:**
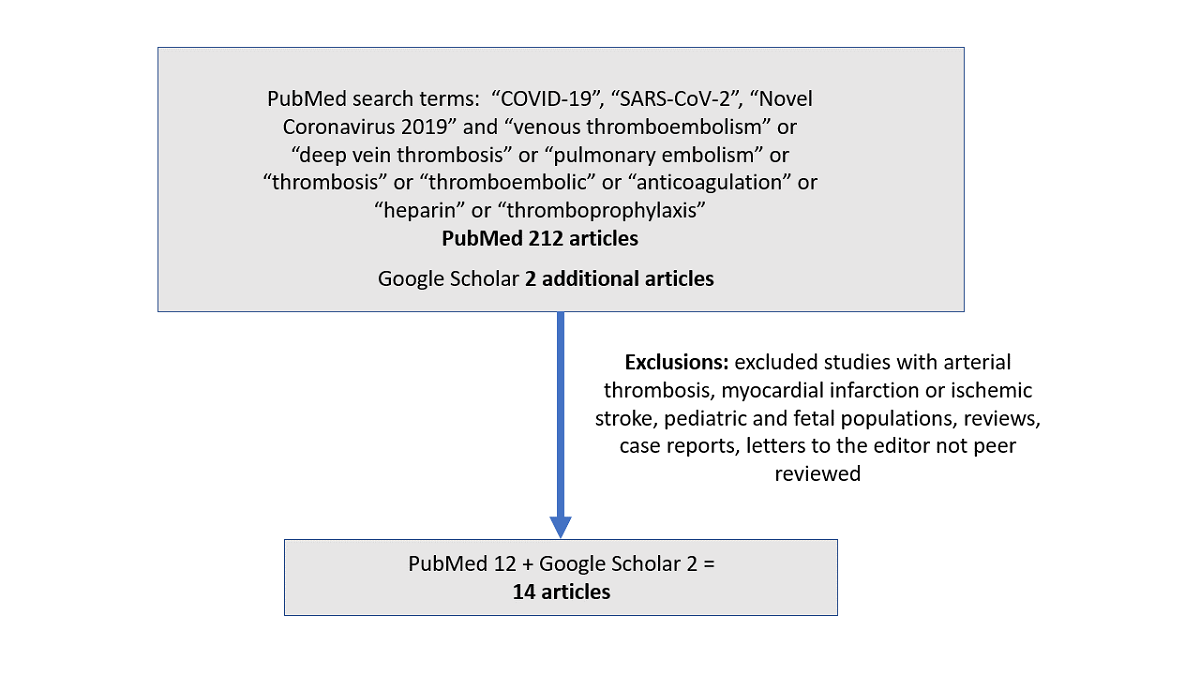
Study selection flowchart showing inclusion and exclusion criteria.

The initial PubMed literature review returned 212 journal articles, of which 12 studies were included in our review. The supplementary Google Scholar and journal article references search identified an additional 2 studies. In total, 14 studies were included in our review [6,9,13-24] (Figure 1).

Studies included were observational (Table 1) and predominantly based on experience at a single center (single center: n=10; multicenter: n=4). The total patient sample size was 1677 (range 26-388) and represented a multinational patient population (China: n=272; France: n=206; Italy: n=415; Netherlands: n=382; Spain: n=156; United States: n=44). The weighted median study duration was 37.2 (SD 17.4) days and 3 studies reported a median length of stay (LOS) (weighted mean LOS 9.5 [SD 1.8 ]days) with patients receiving both ICU and non-ICU levels of care (Table 2). Five studies (n=352) did not report the patient status (eg, discharged alive, expired, or admitted) at completion of the study period. For the other 9 studies, the designations and counts were as follows: nonsurvivors (n=244), discharged alive (n=717), admitted (n=369), and unknown (n=20).

**Table 1:** Summary of included studies.

Study	Study design	Oxford level of evidence	Sample size, N	Study duration (days)
Lodigiani et al [13]	Prospective cohort	3	388	57
Llitjos et al [14]	Retrospective historical cohort	4	26	23
Wright et al [15]	Retrospective historical cohort	4	44	29
Artifoni et al [16]	Retrospective historical cohort	4	71	16
Poissy et al [17]	Case series	4	107	33
Faggiano et al [18]	Retrospective historical cohort	4	25	14
Demelo-Rodríguez et al [19]	Prospective cohort	4	156	14
Zhang et al [20]	Cross-sectional cohort	4	143	31
Helms et al [21]	Prospective cohort with historical controls	3	150	28
Voicu et al [22]	Prospective cohort	4	56	21
Ren et al [23]	Cross-sectional cohort	4	48	2
Middeldorp et al [6]	Prospective cohort	4	198	59
Cui et al [24]	Retrospective historical cohort	4	81	52
Klok et al [9]	Retrospective historical cohort	4	184	29

**Table 2:** Summary of patient clinical characteristics and factors.

Variable	Studies reporting variable	Sample size, N	Weighted mean (SD)
Age (years)	12	1514	64.2 (3.2)
Male	13	1570	66.8 (9.4)^a^
Female	13	1570	31.2 (10.0)^a^
BMI (kg/m^2^)	4	339	27.7 (1.2)
BMI (>30 kg/m^2^)	1	362	24.1 (0)
D-dimer (ug/mL)	11	998	2.1 (0.9)
Fibrinogen (mg/dL)	6	395	628.4 (118.3)
Prothrombin time (sec)	3	268	14.4 (0.9)
Platelets (x10^9^/L)	8	869	232 (21.3)
SOFA^b^ score	4	291	5.9 (3.1)
History of VTE^c^	6	1061	8.0 (4.5)^a^
Pre-existing anticoagulation	4	796	25.0 (8.1)^a^
ICU^d^ level of care	14	1677	75.8 (53.4)^a^
Invasive mechanical ventilation	5	446	76.5 (55.8)^a^

^a^Expressed as weighted percentages.

^b^SOFA: sequential organ failure assessment.

^c^VTE: venous thromboembolism.

^d^ICU: intensive care unit.

In total, 13 studies reported utilization of VTE chemoprophylaxis or treatment anticoagulation. Most patients (n=1306, 82.4%) received anticoagulation; 17.6% (n=279) did not. Of the patients who received anticoagulation, standard dose VTE prophylaxis was most common (n=691, 52.9%). Patients were also prescribed high dose VTE prophylaxis (n=84, 6.4%) or treatment anticoagulation (n=197, 15.1%). In 25.6% (n=334) of patients prescribed anticoagulation, the dosage or intensity was not specified. VTE diagnosis was determined by systematic screening in 7 studies, 1 of which also implemented systematic screening for PE. For 7 other studies, VTE was diagnosed by usual practice. Three studies exclusively screened for DVT and did not report PE.

**Figure 2:**
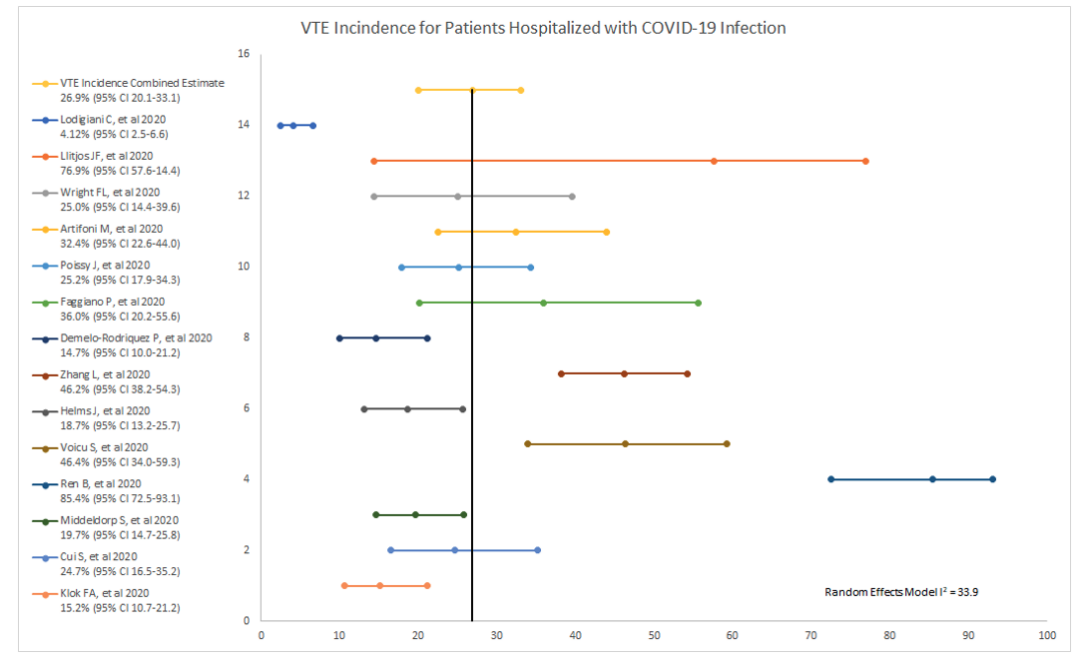
Forest plot of the summary estimate of venous thromboembolism (VTE) incidence.

The combined estimate of VTE incidence was 26.9% (SE 3.1; 95% CI 20.8-33.1) (Figure 2). Occurrence of VTE (n=377) was more often attributed to DVT (n=262) and less often to PE (n=116). The combined estimate of mortality incidence was 24.4% (SE 7.1; 95% CI 10.5-38.2). Absolute values were 244 for nonsurvivors, 717 for discharged alive, 369 for admitted, and 20 for unknown.

Systematic screening for VTE (r^2^=.34, *P*=.03) and study duration (r^2^=–.33, *P*=.03) were both correlated with VTE incidence. There were no associations with VTE and mortality, percentage of patients prescribed anticoagulation, gender, age, or D-dimer level. Multivariate linear regression for the intensity of VTE prophylaxis and VTE incidence was not significant (r^2^=.64; F=.25) nor was a model that included the percentage of patients prescribed VTE prophylaxis or anticoagulation, the percentage of patients in the ICU, gender, age, D-dimer level, study duration, and implementation of systematic screening for VTE (r^2^=.67; F=.58).

**Figure 3:**
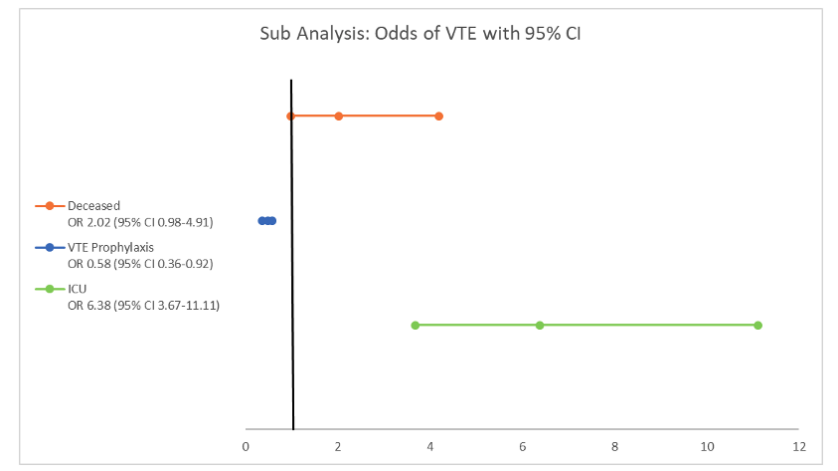
Forest plot of venous thromboembolism (VTE) with anticoagulation and intensive care unit (ICU) admission. OR: odds ratio.

Five studies compared clinical characteristics and outcomes for patients with VTE (n=157) to patients without VTE (n=296). D-dimer was significantly increased in patients with VTE compared to patients without VTE (5.62 [SD 0.9] vs 1.43 [SD 0.6]; *P*≤.001). VTE was decreased in patients receiving anticoagulation (either VTE prophylaxis or treatment anticoagulation) (OR .58, 95% CI .36-.92; *P*=.02) and was increased in patients receiving an ICU level of care during their admission (OR 6.38, 95% CI 3.67-1.11; *P*≤.001). There was no difference in VTE rates for nonsurvivors compared to survivors (OR 2.02, 95% CI .98-4.19; *P*=.06) (Figure 3).

## Discussion

### Principal Findings

In this review, we identified and evaluated 14 studies to assess the incidence of VTE in hospitalized patients with COVID-19 [10]. Our estimated VTE incidence of 26.9% is higher than what has been previously described in the placebo arms (VTE 5%-15%) of clinical trials that evaluated VTE prophylaxis in medically ill patients, as well as in French controls admitted to the ICU with acute respiratory distress syndrome (4.8%), influenza (7.5%) or any cause (6.1%) [2,21]. One explanation for this finding may be the consistently elevated coagulopathy associated with COVID-19 infection [1-3]. Although all 14 studies reported VTE incidence, there was variation in sample size, study duration, hospital LOS, level of care, and VTE prophylaxis and diagnostic strategies. Risk factors for VTE were not reported. This may explain the degree of variation among individual studies around the summary estimate as well as the differences in degree of weighted impact. Further, more than a quarter of the patients included in our systematic review were still hospitalized at the time of study completion. Without including the entire duration of hospitalization for COVID-19 patients, the VTE incidence we report may be an underestimate.

Although the patient populations we evaluated encompassed Asia, Europe, and North America, demographics and clinical characteristics were similar and consistent with previous reports for patients with COVID-19 infection [10]. Accordingly, a high proportion of patients were candidates for anticoagulation and received anticoagulation during their hospitalization. Although the odds of VTE were lower in patients who received anticoagulation, there was no observed association between VTE incidence and anticoagulation intensity. Our review supports the increasingly standard practice of prescribing VTE prophylaxis for hospitalized COVID-19 patients [1,2]. However, future studies will be needed to guide recommendations for the optimal VTE prophylaxis strategy in this cohort of patients. Further research into the pathophysiology of hypercoagulability in these patients may also better inform the most optimal prophylaxis strategy. 

In our review, systematic screening for VTE was correlated with VTE incidence. However, the clinical significance of positive studies is unknown (eg, asymptomatic venous clot or superficial venous clot). Study duration was negatively correlated with VTE incidence, which was not expected, likely reflecting heterogeneity in studied patient populations, including severity of illness, LOS, prevalence of VTE risk factors, and anticoagulation and diagnostic strategies.

Five studies reported data for patients with VTE compared to patients without VTE. The D-dimer level was significantly elevated in patients with VTE, which is consistent with previous studies and supports the prognostic value of D-dimer as a serum biomarker for assessing VTE risk in a hospitalized patient with COVID-19 [1,2]. Additionally, the odds of VTE were higher in patients in the ICU and lower in patients on anticoagulation. However, these studies did not specify the intensity of VTE prophylaxis or dosage. No other patient subgroups who may be at increased VTE risk (eg, comorbidities, pregnancy) were identified. There was no difference in mortality in these subgroups of patients, and therefore the impact of VTE on survival remains unknown and likely confounded by differences in patient populations.

The strengths of our analysis are the inclusion of populations from across the globe, overall sample size, and consistency among studies in reporting data for VTE incidence, level of care, and strategies for VTE prophylaxis as well as diagnostic strategy.

There are several noteworthy limitations in our study. First, the studies included in our analysis are observational. Given that there are no randomized clinical trials at this time, it is difficult to eliminate confounding variables when assessing VTE and clinical associations. Furthermore, the included studies are heterogeneous with respect to reporting of patient demographics, clinical characteristics, method of VTE diagnosis, and strategy for anticoagulation. These studies suggest increased incidence of late or delayed VTE risk [6]. Further, the studies did not consistently report duration of illness, severity of illness, hospital LOS, risk factors for VTE, or presence of other non-VTE indications for anticoagulation. Most importantly, a significant proportion of patients included in our analysis were still hospitalized at the time the study was completed.

Future potential areas of research include arterial thrombosis, hypercoagulability risk factors, occurrence of late-term thrombosis post discharge, and the presence of long-term coagulopathy. As worldwide cases continue to surge, VTE risk in nonhospitalized patients with less acute and nonpneumonia COVID-19 infection also warrant further investigation. Wearable technology is actively being investigated to monitor COVID-19 infection in the community and at home. Examples include the DETECT Health Study, the COVIDENTIFY Study, and the TemPredict Study. A proposed framework exists to develop novel clinical indications for wearable technology, such as early detection of VTE in ambulatory patients based on potential physiologic or wearable markers that could signal increased VTE risk or association. However, feasibility studies would need to be conducted to validate novel use cases [25].

### Conclusion

Coagulopathy associated with COVID-19 infection has emerged as a contributor to morbidity among patients infected with SARS-CoV-2. Early reports demonstrate a significantly increased incidence of VTE. We performed a systematic review to estimate the observed incidence of hospitalized VTE patients with COVID-19 infection. Despite utilization of background VTE prophylaxis and anticoagulation, VTE incidence is historically high. Future studies will provide additional data and generate insights to guide therapeutic decision making and optimize VTE prophylaxis and diagnostic strategies.

## Abbreviations

aPTT: activated partial thromboplastin time
CRP: C-reactive protein
DVT: deep vein thrombosis
ICU: intensive care unit
IL-6: interleukin-6
INR: international normalized ratio
LOS: length of stay
OR: odds ratio
PE: pulmonary embolism
PT: prothrombin time
ULN: upper limit of normal
VTE: venous thromboembolism
